# Depression, anxiety, and stress among college students: a Kashmir-based epidemiological study

**DOI:** 10.3389/fpsyt.2025.1633452

**Published:** 2025-09-10

**Authors:** Amrit Sudershan, Sumaira Rehman, Tafazul Manzoor, Basharat Shaban, Seerat Sultan, Agar Chander Pushap, Srishty Sudershan, Mehraj Bashir, Showkat Ahmad Malik

**Affiliations:** ^1^ Department of Human Genetics, Sri Pratap College, Cluster University Srinagar, Srinagar, Jammu & Kashmir, India; ^2^ Department of Zoology, Sri Pratap College, Cluster University Srinagar, Srinagar, Jammu & Kashmir, India; ^3^ Department of Environmental Science, Sharda University, Delhi, India; ^4^ Department of Education, Dakshina Bharat Hindi Prachar Sabha, Madras, Chennai, Tamil, India; ^5^ Department of Zoology, Central University of Jammu, Samba, Jammu, Jammu & Kashmir, India

**Keywords:** depression, anxiety, kashmiri population, prevalence, epidemiological study

## Abstract

**Introduction:**

Depression and anxiety are major public health concerns, especially among young adults. However, limited regional data exist from Kashmir, Northern India, to guide targeted mental health strategies. Therefore, the present study aimed to assess the levels of depression and anxiety among college-going students in Kashmir and examine associated demographic factors.

**Methods:**

A cross-sectional survey was conducted from January to April 2024, involving 1,471 college students aged 18 to 26 years from institutions across the Kashmir division. Data were collected using an online Google Form after obtaining informed consent. Depression and anxiety were measured using validated scales: PHQ-9 and GAD-7. Statistical analyses included t-tests, chi-square tests, logistic regression, and Pearson correlation to explore associations and trends.

**Results:**

Among the participants, 12.5% exhibited severe depression, with a slightly higher prevalence in females (13.39%). Severe anxiety was reported by 24.26% of students, and 19.17% reported high perceived stress levels. Gender showed notable relevance with anxiety (OR ≈ 1.68, p = 0.0001) and stress (OR ≈ 1.65, p = 0.0007). Correlation analysis revealed positive relationships between depression and stress score (r = 0.6322), anxiety and stress score (r = 0.6824), and depression and anxiety (r = 0.8064), suggesting that higher stress levels correlate with increased depression and anxiety among surveyed individuals.

**Discussion:**

The high prevalence of depression, anxiety, and stress among college students, especially among females, highlights an urgent need for gender-sensitive, campus-based mental health interventions. The strong interrelations among these psychological conditions suggest that comprehensive stress-reduction programs may effectively mitigate multiple mental health risks. Future research should focus on designing and evaluating such targeted strategies to enhance student well-being.

## Graphical Abstract

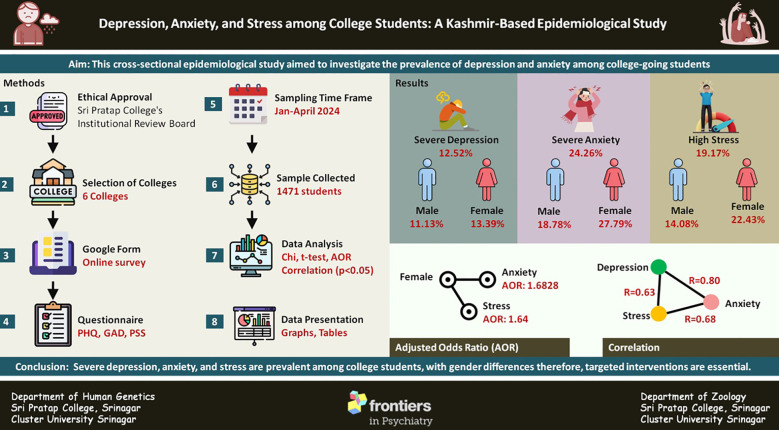

## Introduction

A nation’s progress is closely linked to the well-being of its citizens, as those who are mentally healthy are more likely to make a positive impact, engage in meaningful work, and maintain strong social connections (1). However, a significant barrier to mental well-being is Major Depressive Disorder (MDD), which is characterized by persistent symptoms such as feelings of hopelessness, difficulty concentrating, disrupted sleep, low energy, changes in appetite, and, in severe cases, suicidal thoughts or attempts. These symptoms typically last for at least two weeks and occur nearly every day, significantly impairing an individual’s ability to function (Depression-World Health Organization). According to the data of Global Burden of Diseases (GBD) 2019 identified depression and anxiety were identified as the two most disabling mental health conditions, ranking among the top 25 causes of disease burden worldwide. Around 280 million people globally suffer from depression, representing 3.8% of the population, with higher rates in adults (5.0%) and those over 60 (5.7%). The highest prevalence rates are found in Greece, Palestine, and Spain (Institute for Health Metrics and Evaluation). In India, states such as Tamil Nadu, Haryana, Andhra Pradesh, and Maharashtra report notable depression rates, with Tamil Nadu having the highest at 4.45% (Institute for Health Metrics and Evaluation | (healthdata.org).

Although adults and older populations show higher global prevalence, it is critical to study the mental health of college-going students, as they undergo significant developmental and psychosocial transitions that can heighten vulnerability to psychological distress. Academic pressure, career uncertainty, identity exploration, and changing social environments contribute to an increased risk of mental health issues in this age group (2, 3). Moreover, early identification and intervention in this formative period can prevent long-term psychological morbidity. In the Kashmir region, multiple studies have explored depression and anxiety prevalence (4–6), though none have specifically targeted the college-going population. Therefore, this study aimed to fill this gap by examining the prevalence of depression, anxiety, and perceived stress among college students in Kashmir, making it, to our knowledge, the first of its kind in the region.

## Methodology

### Ethical considerations and consent to participate

This study was conducted in strict adherence to ethical standards. The research methodology was reviewed and approved by the Institutional Ethical Committee (ICE) of Sri Pratap College, Srinagar, Cluster University Srinagar, and Kashmir.

Before participation, each study participant provided informed written consent electronically via an e-form or Google Form, outlining the study’s purpose, the voluntary nature of participation, and the confidentiality of their information. Data collection commenced only after obtaining informed consent from all participants (*discussed below*), ensuring compliance with ethical principles and regulations concerning research involving human subjects.

### Study design, sampling approach, and sample size estimation

This study employed a cross-sectional design to investigate the prevalence of depression, anxiety, and stress among college students of Kashmiri descent, aged 18–26 years. A two-stage sampling strategy was adopted to balance representativeness and practicality, combining simple random sampling for college selection and convenience sampling for participant recruitment.

Furthermore, the sample size was estimated based on prior knowledge, considering a significance level (α) of 0.05, an expected proportion of 0.25, and a precision (margin of error) of 0.03 (Equation 1). Using these parameters, the minimum required sample size was calculated to be 801 participants.


(1) $$n=\frac{Z^{2}.p.\left(1-p\right)}{d^{2}}\;$$

Where: **n** = required sample size, **Z** = standard normal deviate at 95% confidence level (1.96), **p** = expected proportion (0.25, based on prior knowledge), and d = margin of error (0.03).

### College selection & participant recruitment

In the first stage, a simple random sampling method was used to select colleges from the Kashmir region. A comprehensive list of all colleges in the region was compiled using Excel. The “RAND () function” in Excel was employed to randomly select six colleges from this list. Random sampling was chosen at this stage to ensure that every college had an equal chance of being selected, thereby minimizing selection bias and enhancing the representativeness of the sample. The decision to select six colleges was made to ensure the study maintained a manageable scope, given the logistical constraints and the available research resources. This number allowed for adequate representation of the student population while enabling in-depth analysis within a feasible timeline.

In the second stage, a convenience sampling method was used to recruit participants from the selected colleges. Convenience sampling was deemed appropriate at this stage due to the practical challenges of accessing students directly and the necessity of ensuring a sufficient sample size within the limited data collection period (January 2024 to April 2024). Furthermore, the online nature of data collection facilitated by Google Forms inherently lends itself to convenience sampling, as participants self-selected based on their willingness and availability to complete the survey. To minimize potential selection bias introduced by convenience sampling, we recruited participants from multiple colleges across various academic disciplines to improve the diversity and representativeness of the sample.

### Data collection procedure

To ensure participant confidentiality and voluntary participation, data were collected via an online Google Form designed with a clear and sequential structure. The form was disseminated through official college communication channels and student groups, such as WhatsApp groups, accompanied by a briefing that explained the study’s objectives and significance. The online format allowed for greater reach, ease of participation, and flexibility for respondents. The Google Form followed a three-step structure: (a): Introductory Section: Participants were presented with an introductory page detailing the purpose of the research and requesting their consent to participate. Only those who provided informed consent could proceed. (b): Demographic Information Section: Participants were required to provide basic demographic details, including age, gender, and educational background. (c): Diagnostic Section: This section featured standardized questionnaires to assess depression, anxiety, and stress (*see Diagnostic Procedure*). The strategic use of a Google Form ensured participant anonymity and confidentiality while also fostering transparency and trust in the research process. To ensure data security and prevent duplicate responses, the “Limit to 1 response” feature in Google Forms was activated, allowing each participant to submit only one response. [Note: Participants were informed about the study’s purpose and provided consent before proceeding with the survey. Due to the anonymous nature of data collection, withdrawal of data post-submission was not possible.

### Diagnostic procedures

Participants who agreed to take part in the study were assessed for depression, anxiety, and perceived stress using well-established tools. Depression was evaluated using the Patient Health Questionnaire-9 (PHQ-9), a widely recognized instrument designed to screen for and diagnose depressive disorders following DSM-5 criteria (7). Details of the PHQ-9 scoring method can be found in **Supplementary Table 1** of the **Supplementary File**. To assess anxiety, we used the Generalized Anxiety Disorder Assessment (GAD-7), a trusted tool for identifying and diagnosing generalized anxiety disorder (8). The scoring method for the GAD-7 is outlined in **Supplementary Table 2** of the **Supplementary File**. In addition to depression and anxiety, we also assessed perceived stress levels using the Perceived Stress Scale (PSS), a widely accepted measure for evaluating stress perception (NH Dept. of Administrative Services). Information about the PSS scoring method is provided in **Supplementary File** of the **Supplementary File**. All assessments were conducted in English, as all participants were fluent in the language, making it unnecessary to modify the wording of the questionnaires.

### Data cleaning and analysis

Following data collection, a rigorous data-cleaning process was implemented, which was done in Microsoft Excel 2019. Exclusion criteria were established to ensure the integrity and reliability of the dataset. These criteria included (1): obtaining participant consent, (2): restricting participant age to the range of 18–26 years, (3): ensuring completion of the entire survey questionnaire, and (4): confirming that students were from the population of the Kashmir region and not from other areas. Additionally, any responses with missing data were systematically identified and excluded from the final analysis to maintain the accuracy and consistency of the dataset. By applying these criteria, unnecessary, incomplete, or inconsistent responses were identified and removed, ensuring the integrity of the dataset. In addition, the responses of the participants were then coded into a numeric form to facilitate analysis in Excel 2019.

### Statistical analysis

The cleaned dataset subsequently underwent comprehensive statistical analysis to explore patterns, trends, and associations related to the prevalence of conditions among college-going students in the Kashmir population (Northern India). Descriptive data analysis of continuous and discrete variables utilized mean ± standard deviation and frequency distribution methods, respectively. To identify significant differences between continuous and discrete variables, t-tests and chi-square analyses were employed, respectively. Adjusted Odds Ratio (AOR) using Multiple Logistic Regression (MLR) was used to determine the association between dependent variables (depression and anxiety) and independent variables (Demographic variables). The Pearson correlation method is used to find the degree of linear relationship between two continuous variables. All calculations were conducted using free online statistical software, specifically the Chi-Square Calculator (2x2-5x5) from Socscistatistics.com, and MedCalc’s statistical software for Logistic Regression and correlation. A significance threshold of p< 0.05 and a 95% confidence interval were considered significant for all statistical tests.

[Note: For binary logistic regression, the response variables such as depression, anxiety, and perceived stress were dichotomized based on standard cut-off scores. For depression, individuals scoring between 15–19 (moderately severe) and 20–27 (severe) were coded as “1” (indicating high depression), while those scoring below this range were coded as “0”. For anxiety, scores of 10–14 (moderate) and 15–21 (severe) were categorized as “1”, and lower scores as “0”. Similarly, for perceived stress, scores between 27–40 were classified as “1” (high stress), while scores below 27 were coded as “0”.

## Result

**Figure 1:**
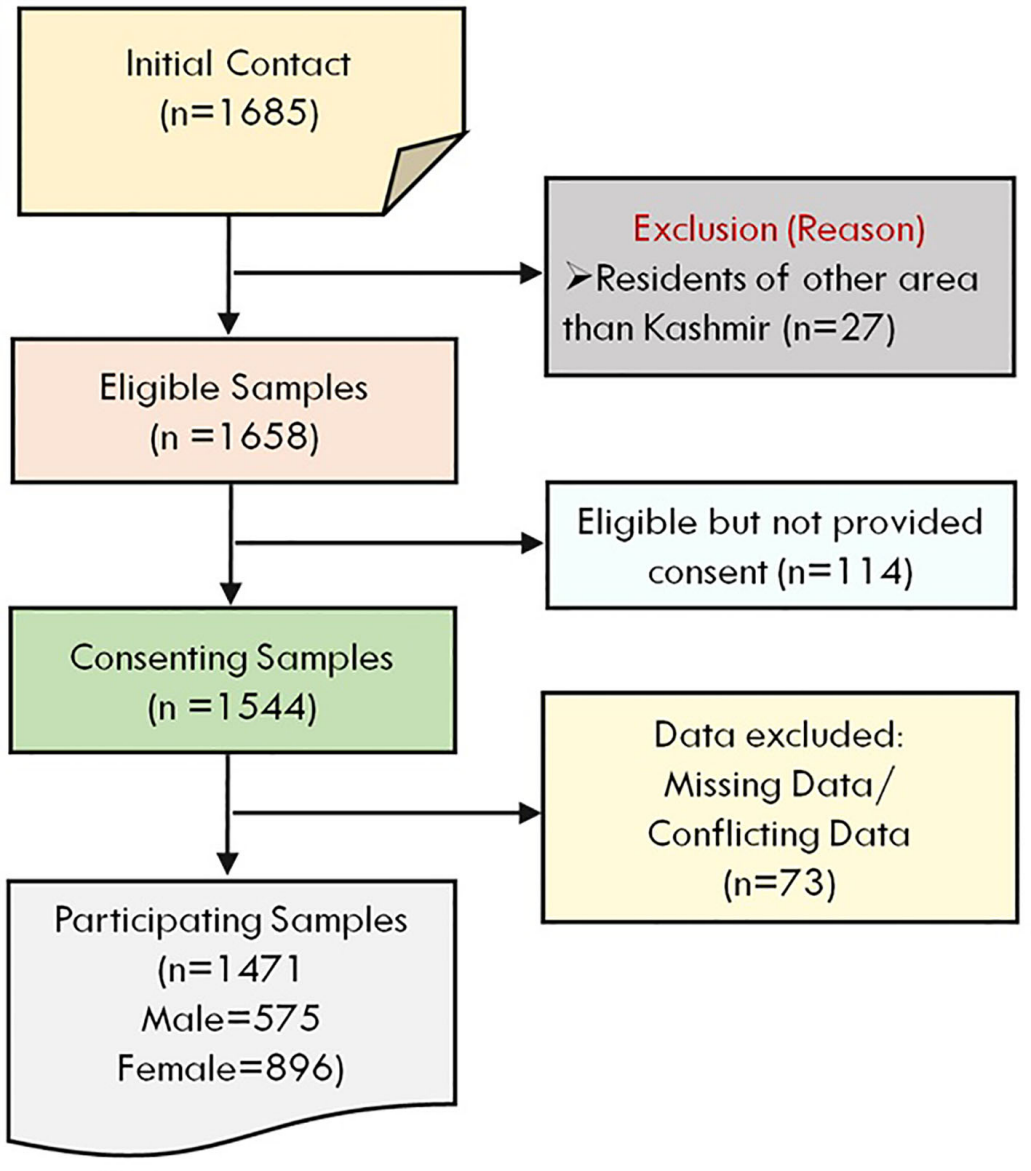
Flowchart illustrates the stepwise selection process of study participants, including exclusions, consent status, and final sample distribution by gender.

**Figure 2:**
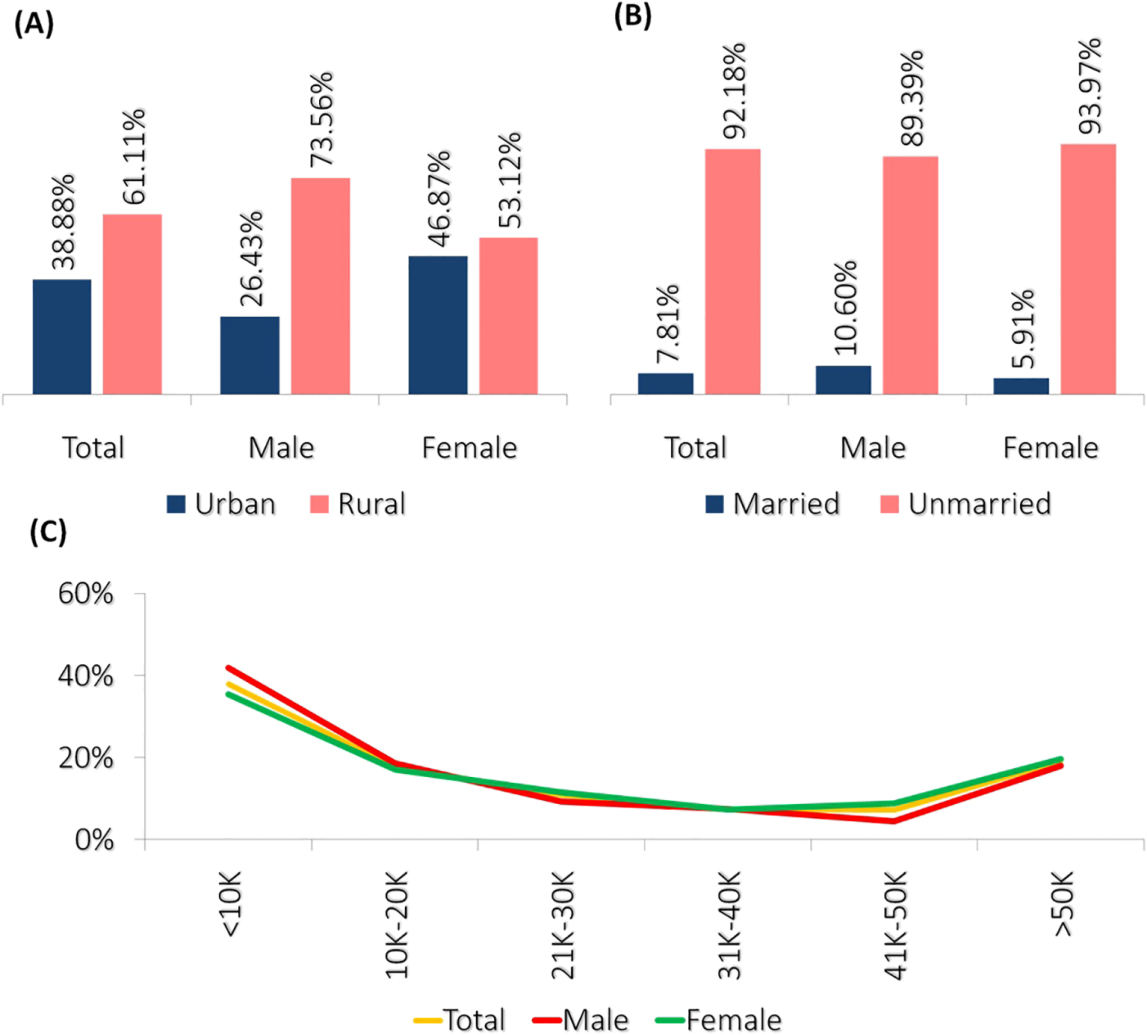
The presented graphs illustrate the demographic characteristics of the surveyed population. **(A)** The bar graph shows the distribution of participants based on their residential location, indicating a higher proportion of rural residents compared to urban residents across total, male, and female groups. **(B)** The bar graph represents the marital status distribution, revealing a significantly higher percentage of unmarried individuals in all categories. **(C)** The line graph depicts the distribution of guardian income levels, showing a declining trend as income levels increase, followed by a slight rise in the highest income category.

In the present investigation, the surveyed population comprises 1471 students (male = 575 and female=896) who were included after excluding a certain number of students (**Figure 1**) using exclusion criteria (*see section:* Data Cleaning and Analysis). The average age of participants was 21.66 ± 1.94 years, with no significant difference between males (21.63 ± 1.94) and females (21.69 ± 1.95; t = 0.577, p = 0.564). The sample was predominantly rural (61.1%), with 38.9% from urban areas (**Figure 2A**). Most participants were unmarried (92.2%) (**Figure 2B**). Gender distribution across rural and urban settings showed a higher proportion of females in urban areas and more males in rural settings. Regarding socioeconomic background, the majority of students’ guardians reported monthly incomes between ₹10,000 and ₹50,000 (**Figure 2C**), with 37.9% earning below ₹10,000 and 19.0% above ₹51,000. A detailed summary of demographic characteristics and gender-based comparisons is presented in **Table 1**.

**Table 1:** Distribution of demographic features among study participants.

Variable	Sub-grouping	Total (n=1471) (%)	Male (n=575) (%)	Female (n=896) (%)
Location	Urban	572 (38.88%)	152 (26.43%)	420 (46.87%)
	Rural	899 (61.11%)	423 (73.56%)	476 (53.12%)
Marital status	Married	115 (7.81%)	61 (10.60%)	53 (5.91%)
	Unmarried	1356 (92.18%)	514 (89.39%)	842 (93.97%)
Religion	Muslim	1404 (95.44%)	552 (96%)	852 (95.08%)
	Hindu	57 (3.87%)	22 (3.82%)	35 (3.90%)
	Sikh	8 (0.54%)	1 (0.17%)	7 (0.78%)
	Buddhist	2 (0.13%)	0 (0%)	2 (0.22%)
Guardian monthly income	<10,000	558 (37.93%)	241 (41.91%)	317 (35.37%)
	10,000-20,000	260 (17.67%)	107 (18.60%)	153 (17.07%)
	21,000-30,000	158 (10.74%)	54 (9.39%)	104 (11.60%)
	31,000-40,000	109 (7.40%)	43 (7.47%)	66 (7.36%)
	41,000-50,000	106 (7.20%)	26 (4.52%)	80 (8.92%)
	Above 51,000	280 (19.03%)	104 (18.08%)	176 (19.64%)

**Figure 3:**
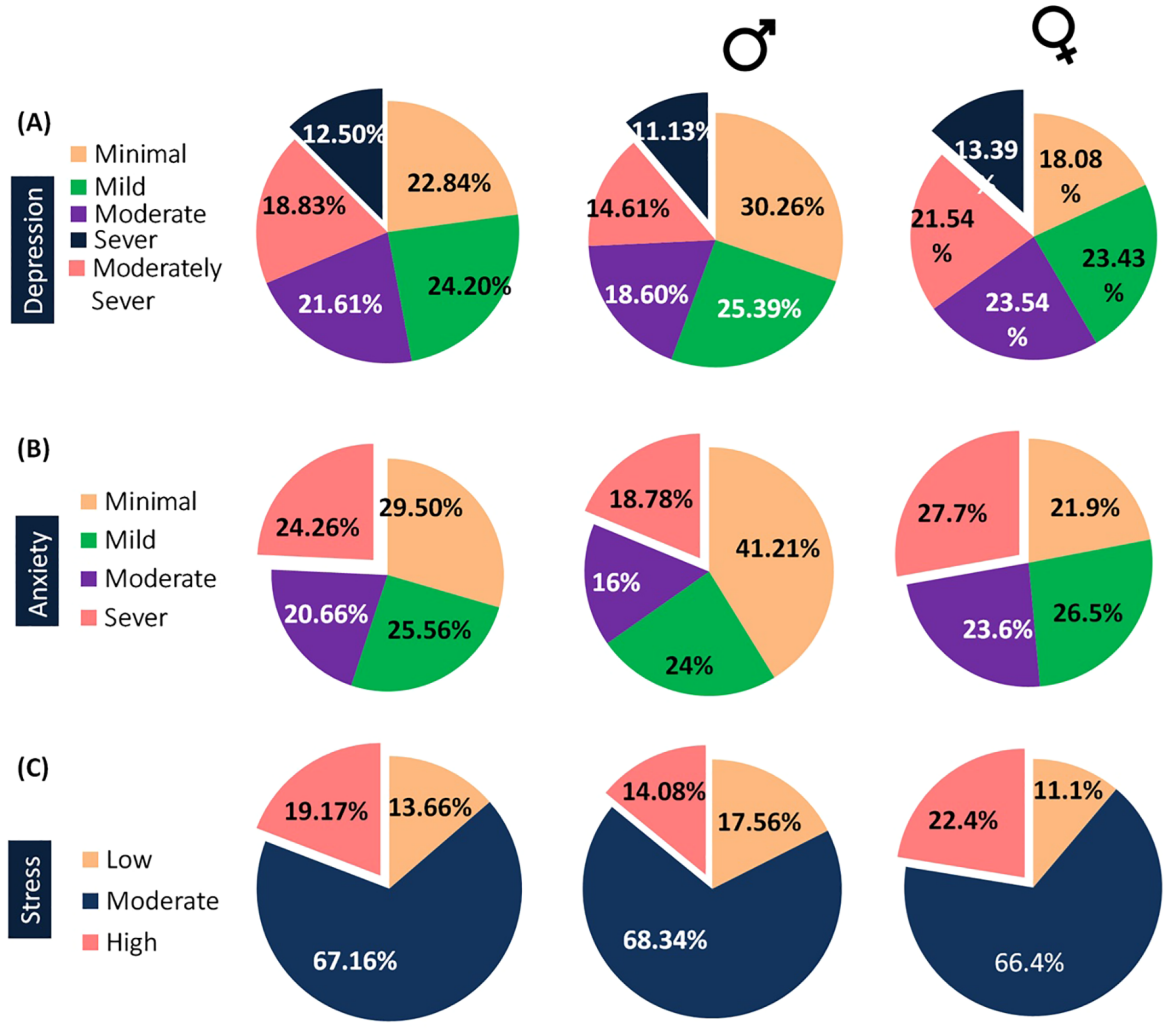
The presented pie charts illustrate the frequency distribution of mental health conditions among the surveyed population. **(A)** The pie charts display the prevalence of depression across the overall, male, and female participants, showing varying proportions across different severity levels. **(B)** The pie charts highlight the distribution of anxiety levels in the total population, as well as by gender. **(C)** The pie charts depict the prevalence of perceived stress among the overall, male, and female groups, revealing distinct trends in stress severity.

After analyzing the demographic features, the prevalence of different conditions among the surveyed students was observed using various questionnaires. Depression levels were assessed using the PHQ questionnaire. It was revealed that minimal depression was observed in 22.84% of the total sample (**Figure 3A**). In contrast, moderately severe depression was identified in 18.83% of the sample. Notably, there was a higher prevalence of moderately severe depression among females (21.54%) compared to males (14.61%). Furthermore, it was observed that severe depression was present in 12.5% (**Figure 3A**) of the total sample, with a slightly elevated prevalence among females (13.39%) compared to males (11.13%) (**Table 2**).

**Table 2:** Prevalence of depression, anxiety, and stress among student of colleges in Kashmir students.

Condition variable	Sub-grouping	Total		Male		Female	
		n=1471	Prevalence [CI]	n=575	Prevalence [CI]	n=896	Prevalence [CI]
Depression (PHQ)	1-4 (Minimal depression)	336	22.84 [20.70-24.70]	174	30.26 [26.51-34.02]	162	18.08 [15.56-20.60]
	5-9 (Mild depression)	356	24.2 [22.01-26.39]	146	25.39 [21.83-28.95]	210	23.43 [20.66-26.21]
	10-14 (Moderate depression)	318	21.61 [19.51-23.72]	107	18.60 [15.43-23.79]	211	23.54 [20.77-26.33]
	15-19 (Moderately severe depression)	277	18.83 [16.83-20.83]	84	14.61 [11.72-17.50]	193	21.54 [18.85-24.23]
	20-27 (Severe depression)	184	12.5 [10.82-14.20]	64	11.13 [8.56-13.70]	120	13.39 [11.16-15.62]
GAD-7 anxiety severity	0–4 (Minimal Anxiety)	434	29.5 [27.17-31.83]	237	41.21 [37.19-45.24]	197	21.98 [19.27-24.70]
	5–9 (Mild Anxiety)	376	25.56 [23.33-27.79]	138	24 [20.51-27.49]	238	26.56 [23.67-29.45]
	10–14 (Moderate Anxiety)	304	20.66 [18.60-22.74]	92	16 [13.00-19.00]	212	23.66 [20.88-26.44]
	15–21(Severe Anxiety)	357	24.26 [22.08-26.46]	108	18.78 [15.59-21.98]	249	27.79 [24.86-30.72]
Perceived Stress Scale	0-13 (Low Stress)	201	13.66 [11.91-15.42]	101	17.56 [14.45-20.68]	100	11.16 [9.10-13.22]
	14-26 (Moderate Stress)	988	67.16 [64.77-69.57]	393	68.34 [64.55-72.15]	595	66.41 [63.31-69.50]
	27-40 (High Perceived Stress)	282	19.17 [17.16-21.18]	81	14.08 [11.24-16.93]	201	22.43 [19.70-25.16]

Regarding the anxiety disorder, insights into varying anxiety levels among surveyed students were provided by the GAD-7 questionnaire. Minimal anxiety was reported by 29.56% (n=434) of the students. Moderate anxiety was noted in 20.66% (n=304), with a higher prevalence among females (23.66%) compared to males (16.00%) (**Figure 3B**). Severe anxiety was experienced by 24.26% (n=357) of students, with a slightly higher prevalence among females (27.79%) compared to males (18.78%) (**Table 2**).

The analysis of the stress among surveyed students using the Perceived Stress Scale unveiled varying stress levels. Among the participants, 201 students (13.66%) reported low stress (0-13), with 101 males (17.56%) and 100 females (11.16%) falling within this category (**Figure 3C**). A majority of students, comprising 988 individuals (67.16%), experienced moderate stress (14-26), including 393 males (68.34%) and 595 females (66.40%). Additionally, 282 students (19.17%) reported high perceived stress (27-40), with 81 males (14.08%) and 201 females (22.43%) experiencing this level of stress (**Table 2**).

**Figure 4:**
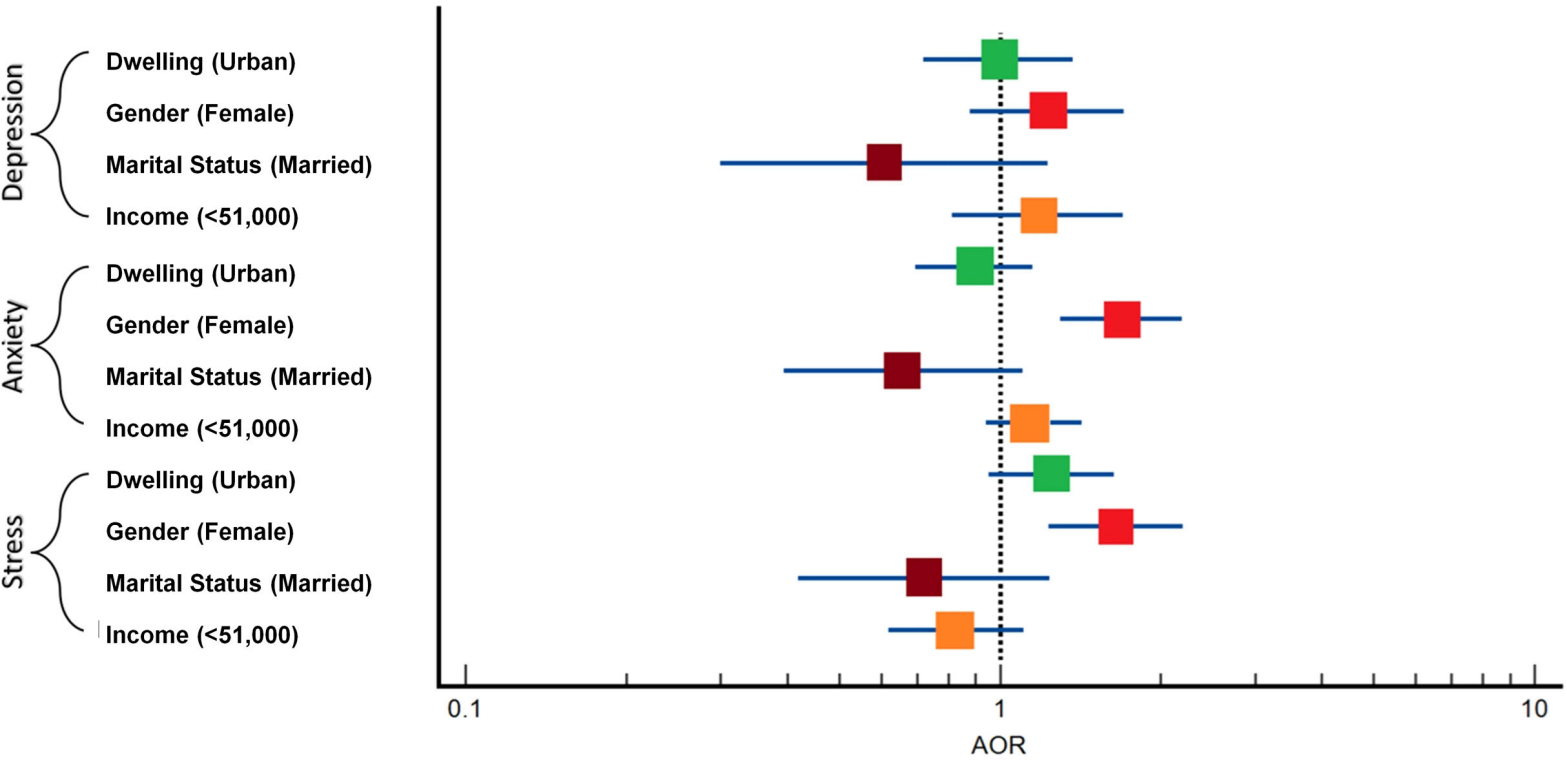
The forest plot shows the association between demographic factors and the risk of depression, anxiety, and stress. Each square represents the Adjusted Odds Ratio (AOR) with confidence intervals for factors such as dwelling place, gender, marital status, and guardian’s income. The dotted line (AOR = 1) indicates no effect.

The analysis of variables linked to mental health outcomes highlighted significant associations (**Table 3**). Gender showed notable relevance with anxiety (OR = 1.68, p = 0.0001) and stress (OR = 1.65, p = 0.0007) (**Figure 4**). Additionally, correlation analysis unveiled positive relationships between depression and stress score (r = 0.6322), anxiety and stress score (r = 0.6824), and depression and anxiety (r = 0.8064), indicating that higher stress levels correlate with increased depression and anxiety among the surveyed individuals (**Table 4**).

**Table 3:** Logistic regression analysis of factors associated with depression, anxiety, and stress among Kashmiri College students.

Out come	Variable	Coefficient	Std. Error	A OR	CI	P-value
Depression	Dwelling Place (Ref.: Rural)	-0.0026	0.1656	0.997	0.720-1.37	0.987
	Gender (Ref.: Male)	0.20281	0.16928	1.224	0.8790 to 1.7068	0.2309
	Marital Status (Ref.: Unmarried)	-0.49828	0.35978	0.6076	0.3002 to 1.229	0.1661
	Monthly income of guardian (Ref.:>51,000)	0.1621	0.18710	1.1761	0.815 to 1.697	0.3861
Anxiety	Dwelling Place (Ref.: Rural)	-0.11141	0.12877	0.8946	0.6950 to 1.1514	0.3869
	Gender (Ref.: Male)	0.52048	0.13369	1.6828	1.2949 to 2.1870	0.0001
	Marital Status (Ref.: Unmarried)	-0.41769	0.26237	0.6586	0.3938 to 1.1014	0.1114
	Monthly income of guardian (Ref.:>51,000)	0.076246	0.14167	1.0792	0.8176 to 1.4246	0.5905
Stress	Dwelling Place (Ref.: Rural)	0.21989	0.13760	1.2459	0.9514 to 1.6316	0.1100
	Gender (Ref.: Male)	0.49853	0.14772	1.6463	1.2324 to 2.199	0.0007
	Marital Status (Ref.: Unmarried)	-0.32875	0.27624	0.7198	0.4189 to 1.2370	0.2340
	Monthly income of guardian (Ref.:>51,000)	-0.18825	0.14859	0.8284	0.6191 to 1.1085	0.2052

AOR, Adjusted Odds Ratio; CI, Confidence Interval; Std. Error, Standard Error.

Legends:

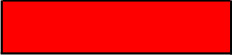
Non-Significant P-value
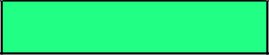
Significant P-value

**Table 4:** Correlation analysis results between depression, anxiety, and stress score.

S. No	Category	Type	Correlation coefficient r	95% CI	P-value
1	Dependent (Y)	Depression	0.6322	0.6005-0.6620	<0.0001
	Independent (X)	Stress Score			
2	Dependent (Y)	Anxiety	0.6824	0.6541-0.7088	<0.0001
	Independent (X)	Stress Score			
3	Dependent (Y)	Depression	0.8064	0.7878-0.8236	<0.0001
	Independent (X)	Anxiety			

Legends:

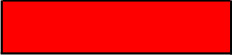
Non-Significant P-value
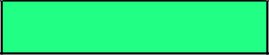
Significant P-value

## Discussion

College life represents a pivotal stage in an individual’s academic and personal development. It serves as a bridge between foundational education and professional or higher academic pursuits, promoting not only knowledge acquisition and skill enhancement but also self-awareness and identity formation. However, this transitional period is often accompanied by considerable psychological stress, which can manifest as mental health concerns, including depression, anxiety, and somatic symptoms such as headaches. Among these, anxiety has been identified as particularly prevalent among college students globally (9). In the context of Kashmir, although mental health issues have been investigated within the general population, there is a noticeable gap in research focusing specifically on college students (4–6, 10–12). Addressing this gap, the present study seeks to assess the prevalence of depression, anxiety, and perceived stress among college students in the region, contributing novel insights to an underexplored area of public mental health research.

After utilizing the cross-sectional study method, the study utilized the PHQ questionnaire to assess depression levels among college students, revealing that 18.81% (Male Vs Female: 14.61% Vs 21.53%) experienced moderately severe depression (scores 15-19) and 12.5% of students reported severe depression (scores 20-27) (**Table 2**). However, the present study found that 12.5% of students reported severe depression, which is somewhat similar to findings from studies conducted in various regions of India and foreign countries (13–20). For instance, in District Amritsar, 13.1% of students reported severe depression (21), while in West Bengal, 6.25% of students experienced severe depression (22). Conversely, some studies have reported contradictory results. For example, in Puducherry, only 0.7% of students were classified as having severe depression, and another 0.7% as having very severe depression (23).

In addition, the present study also revealed that severe depression was slightly more common among female students, with 13.39% affected, compared to 11.13% of male students which was consistent with research indicating higher levels of depression among females compared to males (24–34). Females are more susceptible to mental disorders and report more mental health difficulties than their male counterparts which may be due to certain factors such as academic stress, social pressures, less or no social support, body image, academic performance/stress, peer pressure, professional pressure, financial stress in home, and excessive parental expectations (25, 30, 34, 35). On the other hand, the findings are in contradiction with different studies (22, 23, 36, 37), which found that there is no significant difference between genders. Additionally, some evidence showed that a higher percentage of male students suffered varying degrees of depression than females (35, 38–42). There is a possibility that the gender differences that were found might be attributed to the cultural background of the pupils as well as the socialization process that both genders go through (41).

Anxiety levels, assessed using the GAD-7 questionnaire, indicated that 24.26% of participants reported severe anxiety, with a higher prevalence among females (27.79%) compared to males (18.78%) (**Table 2**). These findings align with several previous studies that have reported elevated anxiety levels among female students (24, 26, 29, 43–46). However, they also contrast with other studies that found higher anxiety levels in males (47) or reported no significant gender differences (36, 48).

Analysis of perceived stress levels showed that 19.17% of students reported high stress, with a higher prevalence observed among females (22.43%) compared to males (14.08%) (**Table 2**). The findings are in line with the research (29, 35, 41, 49–51) but in contrast to others (36, 52–54). Gender was found to be significantly associated with anxiety (OR = 1.68, p = 0.0001) and stress (OR =1.65, p = 0.0007), highlighting differing susceptibilities between males and females (**Table 3**). Furthermore, the study identified positive correlations between depression and stress (r = 0.6322), anxiety and stress (r = 0.6824), and depression and anxiety (r = 0.8064). These correlations suggest that higher stress levels were associated with increased levels of depression and anxiety among the surveyed college students (**Table 4**). The results were found to be consistent with previous studies (55–57); however, several studies present alternative views on the relationship between gender, stress, anxiety, and depression among college students (24, 25, 58).

One might wonder why college students, in particular, experience such high levels of stress. Kashmir has long been a region marked by political instability, prolonged conflict, and socioeconomic uncertainty, factors that have significantly impacted the psychological well-being of its population. Over the past two decades, there has been a notable rise in psychiatric morbidity, with depression emerging as a particularly widespread concern (11). Beyond the broader geopolitical context, several personal, academic, and social factors also contribute to student stress. These include academic overload, personal medical history, family background of chronic or mental illness, and dissatisfaction with body image or overall life satisfaction, all of which have been associated with increased risk of depression and anxiety (16, 59). Poor family relationships have been specifically linked to depressive symptoms. Moreover, predictors of academic stress among adolescent girls include introverted personality traits, religious affiliation, having an illiterate father, and pursuing academic streams such as commerce (60). These findings underscore that student mental health is influenced by a complex interplay of individual, familial, academic, and sociocultural factors. In a conflict-affected setting like Kashmir, such stressors are likely to be intensified, making it essential to examine the mental health of college students within this unique and sensitive context.

### Strengths, limitations & future implications

Concerning the strengths of our study, firstly, to our knowledge, this is the first study to explore the prevalence of stress, anxiety, and depression among students in the Kashmiri population. Secondly, we collected data from a large number of students to accurately determine the prevalence of these conditions. Thirdly, we utilized validated and reliable measurement tools to ensure the accuracy and consistency of our findings.

This study has a few limitations that should be considered. First, we did not analyze differences across academic streams or semesters, which may influence outcomes. Secondly, although standardized tools (PHQ-9, GAD-7, and PSS) were used, reliance on self-reported data may introduce response bias. The sampling approach, combining random college selection with convenience sampling of students, may involve some selection bias, though efforts were made to ensure diversity. Since the study was cross-sectional, it cannot show cause-and-effect relationships. Additionally, online data collection may have introduced minor access or attention biases, and real-time support for distressed participants was not feasible due to anonymity. Finally, no correction for multiple comparisons (e.g., Bonferroni) was applied, which may increase the risk of Type I error.

Despite these limitations, our findings offer valuable insights into the mental health status of college students in Kashmir and highlight directions for future research. Further studies are needed to explore these associations more deeply to determine whether true relationships exist or were missed due to study design. This study emphasizes the urgent need for targeted interventions addressing depression, anxiety, and stress among college students. Implementing comprehensive mental health programs within educational institutions could help reduce these conditions and enhance student well-being. In addition, longitudinal studies are recommended to monitor mental health trends over time, while qualitative research could provide a deeper understanding of students’ lived experiences and coping strategies. Future research should also assess the real-world effectiveness of such interventions and explore additional factors contributing to mental health disparities in this population.

## Conclusion

This study highlights the significant mental health challenges faced by college students in Kashmir, with high prevalence rates of depression, anxiety, and stress. The findings underscore the urgent need for targeted interventions within educational institutions to support student well-being. A concrete recommendation is the implementation of on-campus mental health programs, including regular counseling services, stress management workshops, and peer support initiatives. These efforts should be integrated into the academic curriculum to reduce stigma and promote emotional well-being among students.

## Data Availability

The raw data supporting the conclusions of this article will be made available by the authors, without undue reservation.
