# Supplementary material for: Depression, anxiety, and stress among college students: a Kashmir-based epidemiological study
Source: Front Psychiatry. 2025 Sep 10;16:1633452. doi: 10.3389/fpsyt.2025.1633452 (PMC12457365; doi:10.3389/fpsyt.2025.1633452)
Supplement: Supplementary file 1 [file Table1.docx]

**Table S1:** PHQ (Patient Health Questionnaire) Depression Scoring System

| **Total Score** | **Depression Severity** |
| --- | --- |
| 1-4 | Minimal depression |
| 5-9 | Mild depression |
| 10-14 | Moderate depression |
| 15-19 | Moderately severe depression |
| 20-27 | Severe depression |

Here is the link to download the PHQ-7 questionnaire (<https://adaa.org/sites/default/files/GAD-7_Anxiety-updated_0.pdf>).

**Table S2:** GAD-7 (Generalized Anxiety Disorder) Anxiety Scoring System

| **Total Score** | **Depression Severity** |
| --- | --- |
| 0-4 | Minimal Anxiety |
| 5-9 | Mild Anxiety |
| 10-14 | Moderate Anxiety |
| 15-21 | Severe Anxiety |

Here is the link to download the GAD questionnaire (<https://www.hiv.uw.edu/page/mental-health-screening/gad-7>).

**Table S3: Perceived Stress Scale (PSS) Scoring System**

| **Total Score** | **Depression Severity** |
| --- | --- |
| 0-13 | Low Stress |
| 14-26 | Moderate Stress |
| 27-40 | High Perceived Stress/ High Stress |

**Scoring Method:** Assign scores of **0**, **1**, **2**, and **3** to the response categories:

**0** = Not at all, **1** = several days, **2** = More than half the days, **3** = nearly every day

**Table 3:**

**Scoring Method:**

1. For each question, choose from the following options:

**0** = Never, **1** = Almost never, **2** = Sometimes, **3** = Fairly often, **4** = Very often

1. **Reverse Scoring** (for questions **4**, **5**, **7**, and **8**):

**0 → 4, 1 → 3, 2 → 2, 3 → 1, 4 → 0**

1. **Total Score Calculation:**

Add the scores for all the items after applying reverse scoring where required.

The total score can range from **0** to **40**, with higher scores indicating greater perceived stress.

Here is the link to download the PSS questionnaire ([**https://www.das.nh.gov/wellness/docs/percieved%20stress%20scale.pdf**](https://www.das.nh.gov/wellness/docs/percieved%20stress%20scale.pdf)
